# Patient-Reported Outcome Measures for Post-mastectomy Breast Reconstruction: A Systematic Review of Development and Measurement Properties

**DOI:** 10.1245/s10434-020-08736-8

**Published:** 2020-06-29

**Authors:** C. F. Davies, R. Macefield, K. Avery, J. M. Blazeby, S. Potter

**Affiliations:** 1 Bristol Centre for Surgical Research, Population Health Sciences, Bristol Medical School, Bristol, UK; 2 University Hospitals Bristol Foundation NHS Trust, Bristol, UK; 3 Bristol Breast Care Centre, North Bristol NHS Trust, Bristol, UK

## Abstract

**Background:**

Breast reconstruction (BR) is performed to improve outcomes for patients undergoing mastectomy. A recently developed core outcome set for BR includes six patient-reported outcomes that should be measured and reported in all future studies. It is vital that any instrument used to measure these outcomes as part of a core measurement set be robustly developed and validated so data are reliable and accurate. The aim of this systematic review is to evaluate the development and measurement properties of existing BR patient-reported outcome measures (PROMs) to inform instrument selection for future studies.

**Methods:**

A PRISMA-compliant systematic review of development and validation studies of BR PROMs was conducted to assess their measurement properties. PROMs with adequate content validity were assessed using three steps: (1) the methodological quality of each identified study was assessed using the COSMIN Risk of Bias checklist; (2) criteria were applied for assessing good measurement properties; and (3) evidence was summarized and the quality of evidence assessed using a modified GRADE approach.

**Results:**

Fourteen articles reported the development and measurement properties of six PROMs. Of these, only three (BREAST-Q, BRECON-31, and EORTC QLQ-BRECON-23) were considered to have adequate content validity and proceeded to full evaluation. This showed that all three PROMs had been robustly developed and validated and demonstrated adequate quality.

**Conclusions:**

BREAST-Q, BRECON-31, and EORTC QLQ-BRECON-23 have been well-developed and demonstrate adequate measurement properties. Work with key stakeholders is now needed to generate consensus regarding which PROM should be recommended for inclusion in a core measurement set.

Breast cancer is the most common cancer in women, with over 2 million new cases worldwide in 2018.^1^ In the UK, approximately 40% of women who have surgery for breast cancer undergo mastectomy.^2^ Breast reconstruction is offered to patients to improve body image and quality of life.^3^

Decision-making for BR is complex. There are many types of BR surgery ranging from implant-based procedures to microsurgical free-flaps using tissue from the abdomen, thigh, or buttock. Patients and surgeons need high-quality evidence from well-designed studies to help them make informed decisions about their reconstructive options.

Outcome selection, measurement, and reporting in BR studies, however, is currently heterogeneous and inconsistent.^4^^,^^5^ This means that results of individual studies cannot be meaningfully compared or combined, limiting their value for decision-making. To address this, a core outcome set (COS), a minimum set of outcomes to be measured and reported in all future research and audit studies of BR, has recently been developed. Robust Delphi methodology involving over 300 key stakeholders, including patients and healthcare professionals, was undertaken.^6^ The 11-item COS includes clinical (implant and flap-based complications, major complications, and unplanned surgery), patient-reported (quality of life, normality, emotional and physical well-being, donor-site symptoms/morbidity, and self-esteem), and cosmetic (women’s cosmetic satisfaction) outcome domains.

While a COS is an important step in determining what outcomes should routinely be measured, this does not describe how these key outcome domains should be assessed. The next step in improving the quality and consistency of outcome reporting in BR studies is therefore to develop a core measurement set (CMS), a standard set of instruments to assess the core outcome domains.^7^^–^9 Patient-reported outcomes are particularly important in BR, and it is vital that any patient-reported outcome measure (PROM) recommended for use in future studies be robustly developed and validated for use in this population. The COnsensus-based Standards for the selection of health Measurement INstruments (COSMIN) guideline^10^^,^^11^ is a critical appraisal tool for evaluating the methodological quality of studies reporting the development and measurement properties of health-related measures.^12^ This provides a framework to assess the overall quality of outcome measurement instruments for use in research and clinical practice. The aims of this systematic review are to (1) identify candidate PROMs for each patient-reported outcome domain in the BR COS and (2) to critically appraise, compare, and summarize the quality of studies reporting the development and measurement properties of each PROM using the COSMIN guidelines^11^ to inform selection of PROMs for use in future BR studies and inclusion in a BR CMS.

## Methods

This study was registered on the PROSPERO international register of systematic reviews before the literature search was performed (CRD42017075211).

### Search Strategy and Paper Identification

A systematic search strategy was applied to the OVID versions of MEDLINE (1946–February 2019), EMBASE (1974–February 2019), and PsycINFO (1806–February 2019) to identify articles reporting the development and measurement properties of PROMs developed for and/or validated in women undergoing BR surgery. The search was limited to human studies published in English from database inception up to and including the 26 February 2019. Abstracts and conference reports were excluded due to difficulties evaluating incomplete information. Reference lists of included articles were hand searched for further relevant publications. Duplicate records were excluded.

The search strategy used four broad search terms recommended by COSMIN for performing a systematic review of measurement properties;^11^ These were: (1) the constructs of interest, namely the patient-reported outcome domains included in the BR COS^6^ (self-esteem, normality, quality of life, donor-site problems, emotional and physical well-being, and women’s cosmetic satisfaction), (2) the target population (BR), (3) the comprehensive PROM filter developed by the Patient Reported Outcomes Measurement Group of the University of Oxford,^13^ and (4) the measurement properties filter described by Terwee et al.^14^ The full search strategy is detailed in “Appendix”.

Initial scoping work suggested that few PROMs currently exist that have been developed and/or validated specifically for patients undergoing BR surgery. For this reason, no specific construct for BR were included in the search strategy to avoid suitable instruments being inappropriately excluded.

Titles and abstracts of the remaining citations were screened independently for eligibility by two reviewers (C.D./S.P.) using predetermined inclusion criteria. Any discrepancies were resolved by discussion between the two reviewers. If uncertainty remained, the full text was obtained for further review and discussion. The reference lists of retrieved articles and existing reviews were manually searched to identify additional potentially relevant studies.

### Paper Selection

Full-text original papers published in English reporting the development and/or evaluation of the measurement properties of patient-reported outcome questionnaires in women undergoing BR were eligible for inclusion. Further eligibility criteria included that the questionnaire had to have been developed for patient self-completion, evaluate one of the core patient-reported outcome domains identified in the COS (i.e., health-related quality of life, normality; women’s cosmetic satisfaction; physical well-being, emotional well-being, or self-esteem) to be relevant for inclusion in the CMS, and have been specifically developed for and/or evaluated in female patients aged 18 years or over who had undergone BR. Breast reconstruction was defined as reconstruction of the breast after total mastectomy for invasive or preinvasive breast cancer or risk reduction.

Excluded were studies involving patients (1) with breast cancer in general without specific reference to BR, (2) undergoing breast conserving surgery or partial BR [e.g., with latissimus dorsi (LD) miniflaps or chest wall perforator flaps], and (3) undergoing cosmetic breast surgery only (e.g., reduction or augmentation surgery).

Papers were screened for inclusion independently by two reviewers (S.P./C.D.) using standardized proforma based on predetermined inclusion criteria. In cases of uncertainty, full-text papers were obtained for further evaluation. Uncertainties that remained after full-text review were resolved by discussion with an experienced methodologist (K.A./R.M.). Reasons for exclusion were recorded.

### Data Extraction

Data were extracted onto standardized data extraction proformas. Extracted data included (1) characteristics of PROM instruments, including name of instrument, purpose/objective of study, country of study, recall period, and measurement properties evaluated, (2) PROM instruments assessing each patient-reported outcome domain from the BR COS, including COS item definition, name of PROM instrument, outcome/scales being measured, and number of items per scale, and (3) characteristics of included studies of instruments assessing outcomes in women who had undergone BR, including study author/year, country of study/setting, instrument name, sample size, age (mean), target population, type of RBS performed, and the indication for surgery.

### Data Analysis

#### Selection of PROM Instruments for Full COSMIN Evaluation

Nine measurement properties are included in the COSMIN evaluation.^11^ These included content, structural, cross-cultural and criterion validity, hypothesis testing for construct validity, internal consistency, reliability, measurement error, and responsiveness. Definitions of these properties are provided in Table 1.

**Table 1:** Definitions of measurement properties of instruments assessed by COSMIN guidelines

Measurement property	Definition
Internal consistency	The degree of the interrelatedness among the items; the extent to which scores for patients who have not changed are the same using different sets of items from same instrument
Reliability	The proportion of the total variance in the measurements which is due to “true” differences between patients
Measurement error	The systematic and random error of a patient’s score that is not attributed to true changes in the construct to be measured
Content validity	The degree to which an instrument measures the construct(s) it purports to measure; the degree to which the content of an instrument is an adequate reflection of the construct to be measured
Structural validity	The degree to which the scores of an instrument are an adequate reflection of the dimensionality of the construct to be measured
Hypothesis testing for construct validity	The degree to which the scores of an instrument are consistent with hypotheses (for instance, with regard to internal relationships, relationships to scores of other instruments, or differences between relevant groups) based on the assumption that the instrument validly measures the construct to be measured; item construct validity
Cross-cultural validity	The degree to which the performance of the items on a translated or culturally adapted instrument are an adequate reflection of the performance of the items of the original version of the instrument
Criterion validity	The degree to which the scores of an instrument are an adequate reflection of a “gold standard”
Responsiveness	The ability of an instrument to detect change over time in the construct to be measured; item responsiveness

Definitions as described in COSMIN guidelines manual V1.0, 2018^15^

**Fig. 1:**
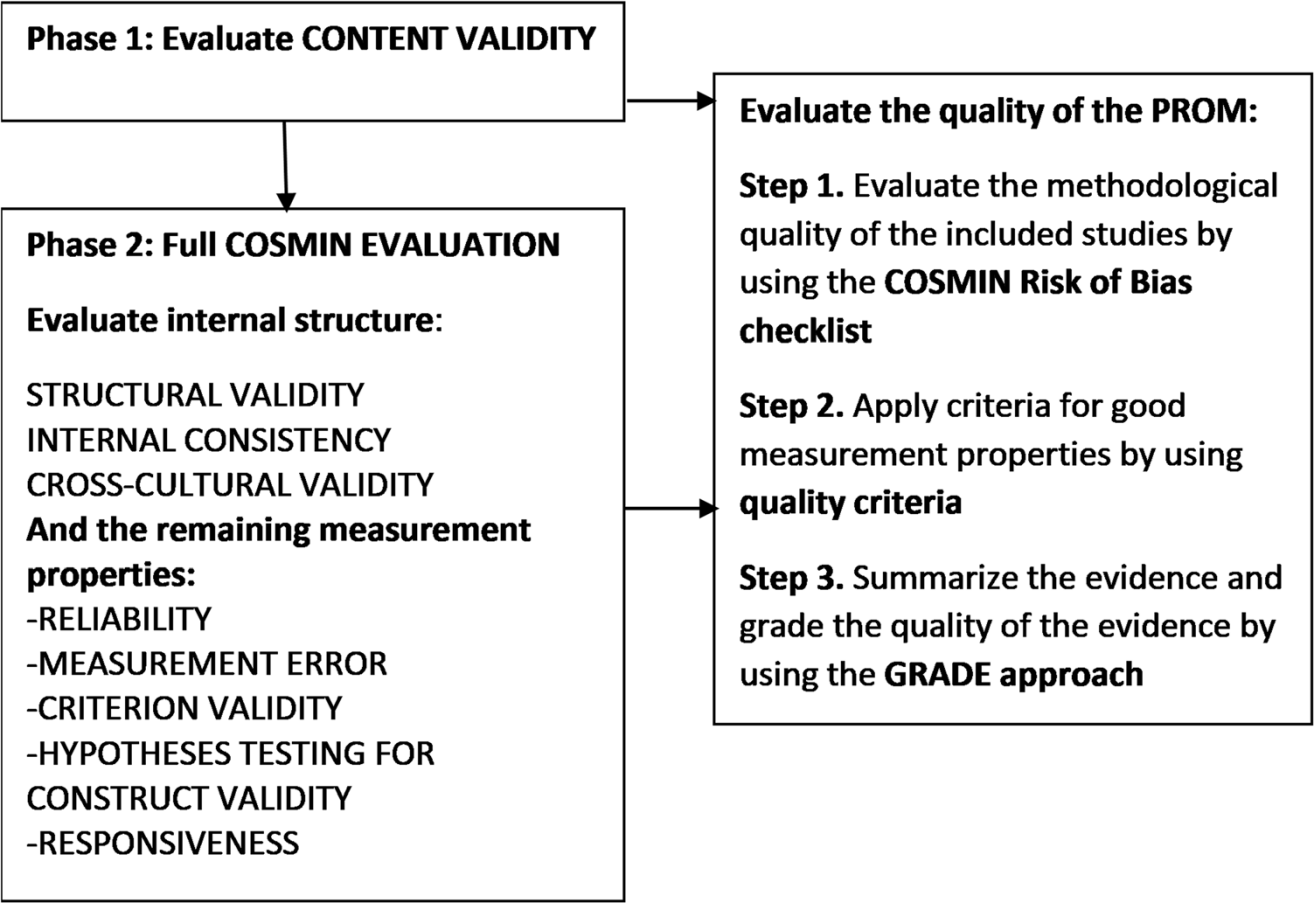
Selecting PROM instruments for full COSMIN evaluation (Phase 1 and 2) (Figure adapted from Mokkink et al.^15^)

Content validity is the most important measurement property of a PROM and refers to whether the content of an instrument appropriately reflects the construct to be measured. It must be clear that items in the PROM are relevant, comprehensive, and comprehensible with respect to the construct of interest and the target population.^15^ Only PROMs assessed by COSMIN criteria as having adequate content validity qualified for full COSMIN evaluation in phase 2 (Fig. 1). PROMs assessed as lacking content validity were excluded from further evaluation (Fig. 1, phase 1).^11^^,^^15^^,^^16^

For PROM instruments undergoing full COSMIN evaluation, data on the instrument’s feasibility were also collected. These included patient comprehensibility, completion time, patient’s required mental and physical ability level, ease of standardization, ease of score calculation, copyright, cost of using instrument, required equipment, and regulatory agency’s requirement for approval.

#### Evaluating Quality of the PROMs

Quality evaluation of the included PROMs consisted of three steps (Fig. 1) and was scored by three reviewers (C.D./R.M./K.A.) independently with disagreements resolved by discussion with a fourth (S.P.).

#### Step 1. COSMIN Risk of Bias Checklist

To evaluate the methodological quality of each single study, the COSMIN Risk of Bias checklist^10^^,^^11^^,^^17^ was used. The COSMIN checklist evaluates the nine measurement properties together with the feasibility and interpretability of the instrument. The risk of bias for each study was rated using a four-point scale as either very good, adequate, doubtful, or inadequate quality and determined by taking the lowest rating of any items (“worst score counts”) within each measurement property.

#### Step 2. Applying Criteria for Good Measurement Properties by Using Quality Criteria

##### 2a: Content Validity

Each result of a single study on PROM development and content validity was rated against the 10 criteria for good content validity.^17^ The results of all available studies were qualitatively summarized to determine whether, overall, the relevance, comprehensiveness, comprehensibility, and overall content validity was sufficient (+), insufficient (−), or indeterminate (?), taking all evidence into account. Studies assessed as having insufficient content validity following this assessment were excluded from further evaluation in the systematic review as these should not be recommended for use.

##### 2b: Remaining Measurement Properties

For instruments assessed as having sufficient content validity, the result of each study for the remaining measurement properties were rated against the criteria for good measurement properties.^11^ Each result was rated as either sufficient (+), insufficient (−), or indeterminate (?).

#### Step 3. Summary of Evidence and Grading of Quality of Evidence

##### 3a: Content Validity

The overall ratings determined in step 2a were also accompanied by a grading for the quality of the evidence using a modified Grading of Recommendations Assessment, Development and Evaluation (GRADE) approach for systematic reviews of clinical trials^18^ (scored as high, moderate, low, or very low). The GRADE approach uses five factors to determine the quality of the evidence: risk of bias, inconsistency, indirectness, imprecision, and publication bias. For evaluating content validity, only three of these factors were applicable, namely risk of bias, inconsistency, and indirectness.

##### 3b: Remaining Measurement Properties

To come to an overall conclusion on the quality of a PROM, the results of all available studies per measurement property had to be consistent. The results were pooled and compared again against the criteria for good measurement properties^11^ to determine whether, overall, the measurement property of the PROM was sufficient (+), insufficient (−), inconsistent (±), or indeterminate (?). As with content validity, quality of the evidence was graded using the GRADE approach for each measurement property. For evaluating measurement properties in systematic review of PROMs, only four of the five factors (as detailed in step 3a above) were taken into account, namely risk of bias, inconsistency, imprecision, and indirectness.

## Results

### Systematic Literature Search

**Fig. 2:**
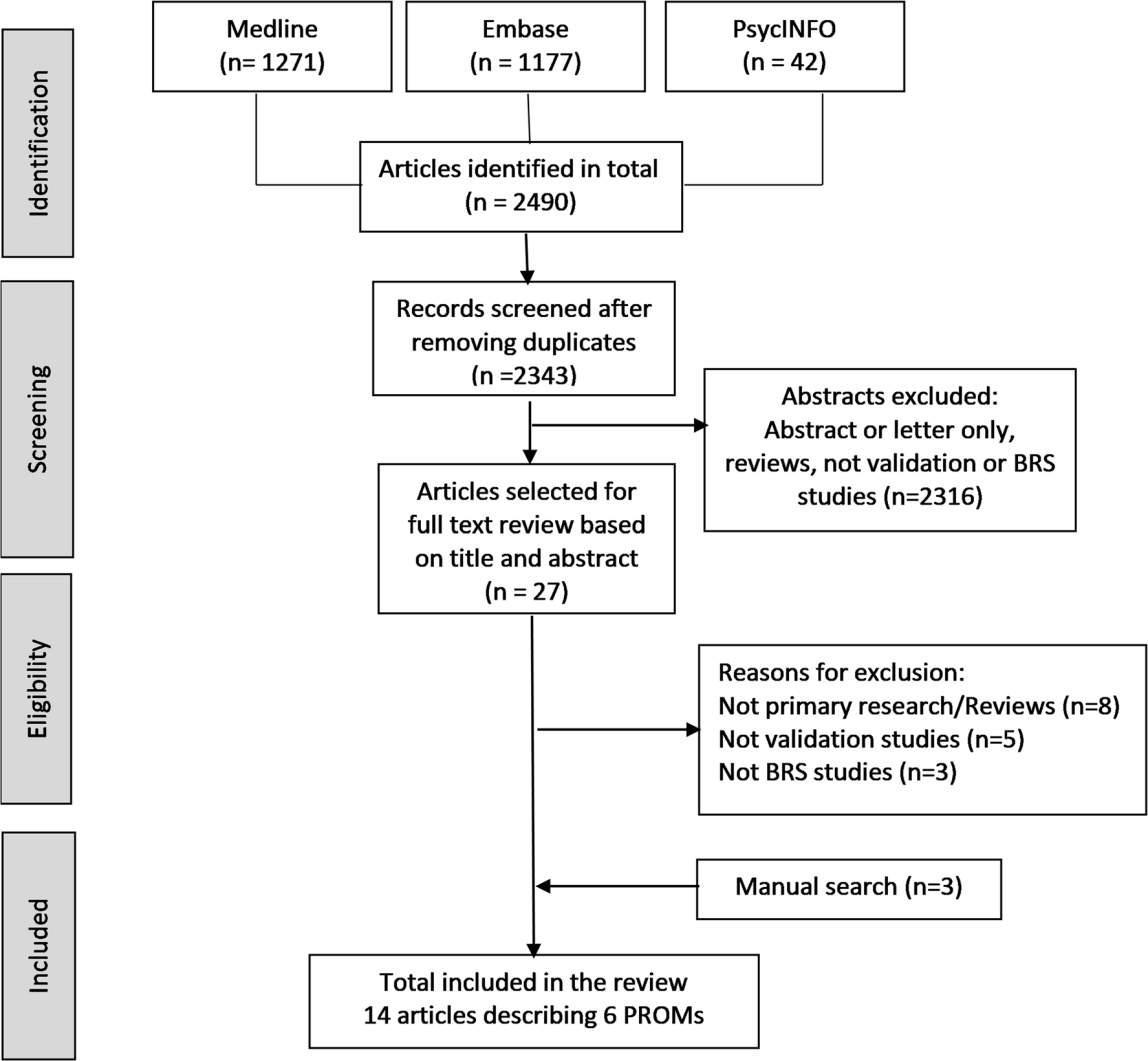
Flow diagram of the systematic review according to PRISMA

After removal of duplicates, 2343 abstracts were screened. For full-text review based on the title and abstract, 27 articles were selected. Of these, 16 articles were excluded from the review for the following reasons: not primary research/reviews (*n* = 8), not validation studies (*n* = 5), or not related to BR surgery (*n* = 3). A further three papers were identified from manual searching. 14 articles describing six BR PROMs met the eligibility criteria and were included in the review (see PRISMA diagram, Fig. 2).

### Characteristics of Included PROMs

Table 2 presents the characteristics of the BR PROMs identified in the review. All included PROMs were evaluated in the English language. The recall period ranged from “within the last week” to “5 years since breast surgery.” Individual studies evaluated different measurement properties and not all measurement properties were assessed for each PROM. A list of which specific measurement properties were measured per instrument is presented in Table 2.

**Table 2:** Characteristics of included PROM instruments and measurement properties evaluated

PROM/instrument (references)	Purpose/objective of study	Country (language in which the questionnaire was evaluated)	Type of admin/recall period	Constructs and subscales measured (no. of items)	COSMIN measurement properties evaluated
BREAST Q (Pusic et al.^19^)	To develop a new patient-reported outcome measure to assess the unique outcome of breast surgery patients	USA and Canada	To comment on their satisfaction or aspects of HRQoL during the previous 2 weeks	Constructs: Satisfaction and HRQoL Three HRQoL domains: physical, psychosocial, and sexual wellbeing. Three satisfaction domains: breasts, outcome, and care (163 items)	Structural validity Acceptability Reliability-internal consistency Test–retest reproducibility Content validity Construct validity (hypothesis testing) Scale validity
BREAST Q (Cano et al.^20^)	To independently validate BREAST Q and focus on the clinical interpretability of the instruments scores	USA and Canada	To comment on their satisfaction or aspects of HRQoL during the previous 2 weeks	Constructs: Breast reduction, augmentation, reconstruction, and mastectomy w/o reconstruction Six domains: satisfaction with breasts, satisfaction with overall outcome, psychosocial well-being, sexual well-being, physical well-being, satisfaction with care	Reliability-internal consistency and test–retest reliability Scale reliability Content validity Structural validity Construct validity (hypothesis testing) Clinical validity
BREAST Q (Browne et al.^21^)	To develop two new measurement scales specifically for LD reconstruction patients to evaluate the esthetic and functional outcomes of LD flap BR and assess their psychometric properties	UK	Outcomes were measured 18 months after surgery	Back appearance scale (8 items) Back and shoulder function scale (11 items)	Structural validity Internal consistency (also measured person separation index)
BREAST Q CAT (Young-Afat et al.^31^)	To develop a computerized adaptive test (CAT) to shorten the BREAST Q’s satisfaction with breasts scale	USA		Constructs: Satisfaction with breasts scale only (of the reconstruction module) Satisfaction with breasts (10–16 items)	Internal consistency
Electronic version of BREAST Q (Fuzesi et al.^32^)	To evaluate the psychometric properties of an electronic version of the BREAST Q in a large online survey	USA		Constructs: QoL, satisfaction, and patient experience (17 items)	Acceptability Reliability (scale) Content validity Construct validity (hypothesis testing) Convergent validity Clinical validity Discriminant validity
BRECON (Temple et al.^22^)	To develop a valid, reliable, and responsive self-admin questionnaire to assess women’s satisfaction with BR	Canada	Average time from surgery to participation was 2.9 years	Constructs: HRQoL and satisfaction (100 items in total)	Content validity (a 100-item pilot questionnaire was developed for further psychometric testing)
BRECON 31 (Temple-Oberle et al.^23^)	To develop a reliable and valid questionnaire to assess patient satisfaction with BR	Canada	Feelings regarding BR within past 2 weeks	Constructs: HRQoL and satisfaction Self-image, arm concerns, intimacy, satisfaction, recovery, self-consciousness, expectations, appearance, nipple, abdomen (31 items) (with additional two subscales where applicable: 4 items for nipple recon and 10 items for abdominal donor site)	Internal consistency Reliability Construct validity Content validity Face validity Criterion validity
BRECON 31 (Temple-Oberle et al.^24^)	To verify the subscale structure of the BRECON 31 using a test sample of women naïve to the questionnaire	Canada	Feelings regarding BR within past 2 weeks	Constructs: HRQoL and satisfaction Self-image, arm concerns, intimacy, satisfaction, recovery, self-consciousness, expectations, appearance, nipple, abdomen (31 items) (with two additional subscales where applicable: 4 items for nipple recon and 10 items for abdominal donor site)	Internal consistency Reliability Content validity Face validity
EORTC QLQ BRECON (Thomson et al.^25^)	To develop and validate the first European multicultural BR specific pre- and post-op PROM	English, Italian, and Swedish translations	3 years since BR (range 1–8 years)	Treatment or surgery related items Body image Sexuality Cosmetic outcome Overall satisfaction (Provisional module: 5 domains and 31 items)	A 31-item pilot questionnaire was developed for psychometric testing
EORTC QLQ-BRECON (BRR) 26 (Winters et al.^26^)	To carry out phase three pretesting of the provisional 31 item EORTC QLQ-BRR questionnaire and assess the relevance, acceptability, and redundancy of Qu/items	English, German, Italian, Swedish, Dutch, and French translations	1–5 years after BR	Provisional three scales (HRQoL): Disease treatment/surgery related symptoms Sexuality Cosmetic outcome (provisional scales were reduced from 31 to 26 items)	Phase III pretesting aimed to assess comprehensibility and comprehensiveness Structural validity Internal consistency Construct validity-convergent validity Discriminant validity
EORTC QLQ-BRECON 23 (Winters et al.^27^)	To carry out phase IV international field-testing of the EORTC BRECON module to finalize scale structure and psychometric testing	International	Standard recall period: the past week	Constructs: HRQoL before and after BR Six subscales: Surgery side-effects, sexuality, satisfaction breast cosmetic, satisfaction nipple cosmetic, satisfaction with surgery, donor site symptoms + single item questions (15 items before mastectomy and BR and 9 items after BR)	Internal consistency Reliability Content validity Structural validity Construct validity (hypothesis testing) Convergent validity Discriminant validity Responsiveness Acceptability Interpretability
MBROS-S (Alderman et al.^28^)	To evaluate the effects of reconstructive technique, procedure timing, and age on esthetic and general satisfaction in women undergoing BR	USA and Canada	1 year after completion of BR	Constructs: HRQoL and satisfaction with breasts Two scales measuring general satisfaction (five items) and esthetic satisfaction (seven items)	Acceptability Internal consistency Reliability Interrater reliability
MBROS-BI (Wilkins et al.^29^)	To evaluate and compare psychosocial outcomes for three common options for mastectomy BR	USA and Canada	Previous 4 weeks	Constructs: HRQoL, and body image and sexual functioning HRQoL: social support and concerns about cancer reoccurrence Body image: appearance in clothes and bathing suit and naked, self-consciousness around others, physical attractiveness, satisfaction with body, self-confidence, self-esteem, and self-consciousness during sexual activity (nine items)	Acceptability Internal consistency Reliability Interrater reliability Responsiveness Validity (comparison with other measures)
Patient-based subjective rating scale for BR appearance (Cohen et al.^30^)	To develop a new instrument for assessing the appearance of autologous BR and compare patient and physician evaluations	USA	Undergone autologous BR at least 6 months previously	Positioning, defects in the breast, the breasts projection, the breast shape, quality of the inframammary fold, quality of the medial contour and overall appearance of breast, and overall satisfaction with BR	Internal consistency Reliability (intraobserver and interobserver) Test–retest reliability

### Assessment of Breast Reconstruction Patient-Reported Outcome Domains

Outcome domains or constructs measured across the identified PROMs included satisfaction with breasts, satisfaction with overall outcome, psychosocial well-being, physical well-being, sexual well-being, health-related quality of life, body image, and sexual functioning. These constructs reflected most of the patient-reported outcome domains in the COS. Two COS constructs, namely normality and self-esteem, were not represented as multiitem domains in the identified PROMs. Several questionnaires, however, included single items relating to each of these constructs. For example, the construct of normality was measured in three PROMs (BREAST-Q, MBROS-BI, and BRECON-31), each of which contained individual items referring to “feeling normal.” For the self-esteem construct, the BREAST-Q included four items in the psychosocial well-being subscale addressing this issue. BRECON-31 included four items addressing self-image and three items relating to feeling self-conscious. Details of domains and PROMs are presented in Table 3.

**Table 3:** Patient-reported outcome domains in reconstructive breast surgery (RBS) core outcome set and instruments/questionnaires evaluating each domain

Relevant patient-reported outcome domains	Core outcome set (COS) item definition	Instruments evaluating patient-reported outcome domain in the RBS COS	
		Name of outcome measurement instrument(s)	Outcome/scales (number of items in scale in brackets)
Donor-site problems/morbidity	Any problems or symptoms arising from the area from which the tissue was taken to reconstruct the breast, including hernias, stiffness, or numbness in the back, tummy, or bottom	BREAST Q:	Abdominal appearance and function (13) Back appearance scale (8) Back and shoulder function scale (11)
		EORTC QLQ-BRECON 23:	Donor-site symptoms (3) Surgical side effects (2) Satisfaction with donor scars (1)
		BRECON 31:	Abdominal donor site (10)
		Electronic version of BREAST Q:	Satisfaction with abdomen (3)
Self-esteem	Feeling self-confident	BREAST Q:	Single items in psychosocial wellbeing subscale: Confident in a social setting (1) Of equal worth to other women (1) Self-confident? (1) Like other women? (1)
		MBROS-BI:	Mental health (5)
		BRECON 31:	Self-image (4) Self-consciousness (3) I feel good about myself (1)
Emotional wellbeing	Feelings of emotional and psychological health after surgery	BREAST Q:	Psychosocial wellbeing (10)
		Electronic version of BREAST Q:	Psychosocial wellbeing (10)
		BRECON 31:	Self-image: item “I feel good about myself” (1)
		MBROS BI:	Role-emotional (3) Mental health (5)
Normality	Feeling “back to normal self” or “whole” as a result of surgery	BREAST Q:	Satisfaction with breasts: How normal do you feel in your clothes? (1) Psychosocial wellbeing: Normal? (1)
		BRECON 31:	Self-image: item “I feel normal” (1)
		MBROS-BI:	Body image: item “I feel whole” (1)
Quality of life	Women’s quality of life following surgery	BREAST Q:	QoL domains: physical, psychosocial, and sexual wellbeing (32)
		Electronic version of BREAST Q:	QoL domains: physical, psychosocial, and sexual wellbeing (32)
		BRECON 31:	Recovery (4)
		EORTC QLQ-BRECON 23:	HRQoL: before mastectomy and BR (4) HRQoL: relevant after BR (15)
		MBROS-BI:	Functional wellbeing (7) Social wellbeing (7)
Physical well-being	Physical activity such as how well women can perform work- and leisure-related tasks after surgery	BREAST Q:	Physical wellbeing: chest and upper body (16) Physical wellbeing: back and shoulder function scale (11)
		Electronic version of BREAST Q:	Physical wellbeing (16) Physical wellbeing (abdomen) (8)
		MBROS-BI:	Vitality (4)
		BRECON 31:	Abdomen strength (5) Abdomen appearance (5) Arm concerns (4) “I have trouble moving my shoulder” (1)
Women’s cosmetic satisfaction	Women’s overall satisfaction with the appearance of their reconstructed breast(s) after surgery	BREAST Q:	Satisfaction with breasts (15) Satisfaction with implants (2) Satisfaction with nipple reconstruction (1)
		BREAST Q CAT:	Satisfaction with breasts (10)
		Electronic version of BREAST Q:	Satisfaction with breast (16) Satisfaction with outcome (7)
		BRECON 31:	Satisfaction (4)
		EORTC QLQ-BRECON 23:	Satisfaction with breast cosmesis (6) Satisfaction with nipple cosmesis (2) Satisfaction with surgery (3)
		MBROS-Satisfaction(S):	General satisfaction with reconstruction (5) Aesthetic satisfaction: breast size/shape/firmness (2)
		MBROS-Body image (BI):	Patient perceptions of physical appearance after BR (9)
		Subjective rating scale for BR:	The overall appearance of the breast (1)

### Characteristics of Included Studies from Systematic  PROMs Review

Table 4 presents the characteristics of the 14 studies included in the review. Studies were largely conducted in North America and/or Canada (*n* = 10), with only three studies based in Europe. One study recruited patients from 28 international centers. The sample sizes ranged from 20 to 5000 women with an age range of 18–84 years and included patients undergoing a range of implant-based and autologous reconstruction, including pedicled and free transverse rectus musculocutaneous (TRAM) flaps and latissimus dorsi reconstruction with and without implants; also, patients undergoing bilateral and unilateral surgery and patients receiving nipple/areola reconstruction as well as nipple-sparing procedures.

**Table 4:** Characteristics of included studies of instruments assessing outcomes in women who have undergone breast reconstruction

Author/year	Country of study/setting	Instrument name	Sample size	Age: mean (SD/range)	Population	Type of BR	Indication for surgery
Pusic et al.^19^	N. America, Canada	BREAST Q	1950 (presurgery = 908, postsurgery = 1807)	Presurgery 43 (14/18–84), Postsurgery 47 (12/18–84)	Pre-op and post-op breast surgery patients recruited from five centers in the USA and Canada	Breast surgery	Breast surgery
Cano et al.^20^	North America	BREAST Q	817	49 (12/20–82)	Breast surgery patients (pre- and post-operative > 18 years)	NR	Breast surgery
Browne et al.^21^	UK	BREAST Q (2 novel scales: back appearance scale and back and shoulder function scale)	1096	Median age = 52 (range 18–50)	Breast cancer patients having latissimus dorsi BR after mastectomy	Latissimus dorsi BR	Breast cancer
Young-Afat et al.^31^	North America	BREAST Q computerized adaptive testing (CAT)	5000	NR (women were 22 years and older)	Women who had undergone implant-based BR randomly selected from 17,000 who had completed the satisfaction-with-breast scale	Implant based BR	Breast cancer
Fuzesi et al.^32^	North America	Electronic version of BREAST Q	1956 (completed BR module)	55 (9.3)	Women with history of BCa completing online survey		Breast surgery
Temple et al.^22^	Canada	BRECON-31	20 (women participating in focus groups)	54 (range 36–69)	Women who had previously undergone BR	16 autologous and 11 alloplastic BR (13 were unilateral & 11 bilateral)	Breast surgery
Temple-Oberle et al.^23^	Canada	BRECON-31	128	52.7	Women who had previously undergone BR	Implant-based BR Abdominal flap BR Combination of an autologous and alloplastic BR	Diagnosed with invasive mammary carcinoma, ductal carcinoma in situ, BRCA-associated or another breast disease
Temple-Oberle et al.^24^	Canada	BRECON-31	50	49.1 (7.6)	Consecutive women presenting for final F/up who had completed BR	Bilateral BR Unilateral BR Implant-based BR Abdominal flap BR Nipple/areola BR	NR
Thomson et al.^25^	UK, Italy, Sweden	EORTC QLQ-BRECON	31 (semistructured interviews)	50 (range 33–66)	LD, TRAM/DIEP, and implant-based BR techniques after mastectomy for BCa	LD, TRAM/DIEAP, and implant-based BR techniques	For women undergoing mastectomy for invasive BCa, ductal carcinoma in situ, or prophylactic surgery
Winters et al.^26^	UK, Austria, Belgium, Italy, and Sweden	EORTC QLQ-(BRR) BRECON 26	150 (retrospective group post BR)	NR	1–5 years after immediate or delayed BR	Mastectomy BR implant only, pedicle LD with implant, autologous LD, and microvascular free abdominal flaps such as TRAM and DIEP	Diagnosed with BCa or ductal carcinoma in situ requiring mastectomy and BR
Winters et al.^27^	28 international centers	EORTC QLQ-BRECON 23	438 (234 in prospective cohort and 204 in cross-sectional cohort). Implants (176), donor-site flaps (166)	50.7 (27–78)	Patients with breast cancer undergoing mastectomy and BR	Mastectomy and BR (implant and autologous BR)	Breast cancer or ductal carcinoma in situ
Alderman et al. 2000	USA and Canada	MBROS-S	212	Implant patients = 48.5 Pedicle TRAM flap = 49.4 Free TRAM flap = 46.4	Mastectomy reconstruction patients: Women who had undergone first time immediate or delayed BR surgery	Expander/implant Pedicle TRAM flap Free TRAM flap	Women who had undergone first time immediate or delayed BR surgery
Wilkins et al.^29^	USA and Canada	MBROS-BI	273	Implant patients = 48.5 Pedicle TRAM flap = 49.4 Free TRAM flap = 46.4	Mastectomy reconstruction patients: Women who had undergone first time immediate or delayed BR surgery	Tissue expander/implant Pedicle TRAM flap Free TRAM flap	Patients undergoing immediate or delayed postmastectomy BR
Cohen et al.^30^	USA	Patient-based subjective rating scale for BR appearance	36	NR	Patients photographs (frontal and lateral views) taken as part of their routine post-op visits after BR	Autologous BR (transverse rectus musculocutaneous flap BR)	NR

*NA* not available, *NR* not reported, *BR* breast reconstruction, *BCa* breast cancer, *TRAM* transverse rectus abdominus myocutaneous flap, *DIEP* deep inferior epigastric artery perforator flap, *LD* latissimus dorsi flap, *F/up* follow-up, *USA* United States; *EORTC QLQ*-*BRR26* European Organization for Research and Treatment of Cancer Quality of Life Questionnaire 26-item breast reconstruction module, *MBROS*-*S* Michigan Breast Reconstruction Outcome study-satisfaction questionnaire, *MBROS*-*BI* Michigan Breast Reconstruction Outcome study-body image questionnaire, *CAT* computerized adaptive testing

### PROM Instruments Selection for Full COSMIN Evaluation

Of the six identified PROM instruments, only three, BREAST-Q,^19^^–^21 BRECON-31^22^^–^24 and EORTC QLQ-BRECON-23^25^^–^27 were considered to have adequate content validity (see below). Of the remainder, the Michigan BR Outcome Study (MBROS) group developed a BR-specific questionnaire item set for satisfaction (MBROS-S)^28^ and body image (MBROS-BI),^29^ using input from an expert panel alone. There was no direct patient input into item generation or reduction, therefore these questionnaires were considered to have insufficient content validity and were excluded from further COSMIN evaluation. Similarly, the patient-based subjective rating scale for BR appearance^30^ did not assess content validity and was excluded. Finally, the BREAST-Q CAT^31^ and the electronic BREAST-Q^32^ were adapted versions of the main BREAST-Q questionnaire. As the main BREAST-Q was being assessed, these were excluded.

### Overall Rating and Grading of Quality of Evidence per Measurement Property for Each PROM

A summary of the analysis and grading of measurement properties for each of the three PROM instruments included for full COSMIN evaluation is presented in Table 5. This includes the summary of pooled results (from each study per PROM), the overall rating, and the grading of the quality of evidence assigned to each of the measurement properties that were measured. The overall ratings and quality of evidence for each measurement property assessed for the three PROMs are presented in a simpler way in Table 6 for ease of comparison between instruments. Cross-cultural validity, measurement invariance, and measurement error were not assessed for any of the three included PROMs and thus are not included in Table 6.

**Table 5:** Summary of findings per measurement property (PROM instruments with “sufficient” content validity only)

COSMIN Measurement property	BREAST Q			BRECON 31			EORTC QLQ-BRECON 23		
	Summary of pooled results	Overall rating	Quality of evidence	Summary of pooled results	Overall rating	Quality of evidence	Summary of pooled results	Overall rating	Quality of evidence
Content validity	Content validity: (+) Relevance: Comprehensiveness: Comprehensibility:	Sufficient (+) Sufficient (+) Sufficient (+) Sufficient (+)	High High High High	Content validity: (+) Relevance: Comprehensiveness: Comprehensibility:	Sufficient (+) Sufficient (+) Sufficient (+) Sufficient (+)	High High High High	Content validity: (+) Relevance: Comprehensiveness: Comprehensibility:	Sufficient (+) Sufficient (+) Sufficient (+) Sufficient (+)	High High High High
Structural validity	Pusic 2009 (+) Cano 2012 (+) Fit to Rasch model was good.	Sufficient (+)	High	Used EFA to assess structural validity	Insufficient (−)	Low	Winters 2014 (?) Winters 2018 (?)	Indeterminate (?)	High
Internal consistency	Pusic 2009 (+) Cronbach’s *α*: 0.81–0.96 Cano 2012 (+) Cronbach’s *α*: > 0.80	Sufficient (+)	High	Temple-Oberle 2012 (?), Cronbach’s *α* ranged from 0.67 to 0.91. Temple-Oberle 2013 (?), Cronbach’s *α* range from 0.34 to 0.92.	Indeterminate (?)	High	Winters 2014 (+) Winters 2018 (+) Cronbach’s *α* > 0.7	Sufficient (+)	High
Cross-cultural validity/measurement invariance	No information available	N/A	N/A	No information available	N/A	N/A	No information available	N/A	N/A
Reliability	Pusic 2009 (+) ICC > 0.70 Cano 2012 (+) ICC = > 0.80	Sufficient (+)	High	Temple-Oberle 2012 (−), ICC > 0.74 Temple-Oberle 2013 (NA)	Insufficient (−)	Very low	Winters 2014 (NA) Winters 2018 (+) Test–retest reliability was good with ICCs for multi item scales ranging from 0.809 to 0.916 and single items from 0.728 to 0.905	Sufficient (+)	Moderate
Measurement error	No test for measurement error	N/A	N/A	No test for measurement error	N/A	N/A	No test for measurement error	N/A	N/A
Criterion validity	No information available	N/A	N/A	Temple-Oberle 2012 Excellent correlation with gold standard (Breast-Q) for satisfaction subscales (PCC = 0.76)	Sufficient (+)	High	No information available	N/A	N/A
Hypothesis testing (for construct validity)	The result was in accordance with the hypothesis	Sufficient (+)	High	The result was in accordance with the hypothesis	Sufficient (+)	Moderate	The result was in accordance with the hypothesis	Sufficient (+)	High
Responsiveness	No information available	N/A	N/A	No information available	N/A	N/A	The result was in accordance with the hypothesis	Sufficient (+)	High

*N/A* not applicable

+ sufficient, − insufficient, ? indeterminate

**Table 6:** Quality of evidence for measurement properties of PROMs

Measurement property^a^	BREAST Q		BRECON 31		EORTC QLQ-BRECON 23	
	Overall rating	Quality of evidence	Overall rating	Quality of evidence	Overall rating	Quality of evidence
	+/−/?	High, moderate, low, very low	+/−/?	High, moderate, low, very low	+/−/?	High, moderate, low, very low
Content validity	+	High	+	High	+	High
*Relevance*	+	High	+	High	+	High
*Comprehensiveness*	+	High	+	High	+	High
*Comprehensibility*	+	High	+	High	+	High
Structural validity	+	High	−	Low	?	High
Internal consistency	+	High	?	High	+	High
Reliability	+	High	−	Very low	+	Moderate
Criterion validity	NA	NA	+	High	NA	NA
Hypothesis testing for construct validity	+	High	+	Moderate	+	High
Responsiveness	NA	NA	NA	NA	+	High

*NA* not assessed/not applicable

^a^Cross-cultural validity, measurement invariance, and measurement error are not listed as these measurement properties were not assessed in any of the three PROMs (BREASTQ, BRECON31, EORTC BRECON23)

### Content Validity

All three included PROMs, BREAST-Q, BRECON-31, and EORTC QLQ-BRECON 23, exhibited sufficient high-quality evidence for the three aspects of content validity (relevance, comprehensiveness, and comprehensibility) as well as the quality of the PROM development, with all three PROMs using extensive input from patients undergoing BR in item formation and from systematic reviews. The development and design of the BREAST-Q questionnaire was extensive, with interviews and focus groups of representative BR patients, and included feedback from healthcare professionals on its relevance and comprehensiveness.

The BRECON-31 used robust item generation and item reduction methods. Item generation was gained from patient focus groups with additional input from an expert panel (plastic surgeons, breast surgeons, and advanced practice nurses) and a literature review. The literature review focused on published articles that related to breast cancer, quality of life, body image, satisfaction, and BR. The EORTC QLQ-BRECON 23 is intended for use alongside the EORTC QLQ-C30 and BR23 to assess patient-reported outcomes in women undergoing mastectomy for invasive breast cancer or ductal carcinoma in situ.^25^ Content validity for this PROM showed sufficient high-quality evidence, with development phases incorporating literature reviews and interviews with patients and healthcare professionals.

### Structural Validity

All three PROMs showed evidence of structural validity. Both BREAST-Q and EORTC QLQ-BRECON 23 were graded “high” for the quality of the evidence. Development of BREAST-Q involved Rasch modeling/methodology (a form of item response theory) to predict individual item responses and evaluate changes in an individual’s health-related quality of life (HRQL).^19^ Results showed that the fit to the Rasch model was good and item locations were spread out (0.7–6.6). EORTC QLQ-BRECON 23 used confirmatory factor analysis to test how well the measured variables represented the number of constructs. Studies included an adequate sample size in the analysis, and this instrument received an overall sufficient rating and high quality of evidence. BRECON-31 used exploratory factor analysis to identify the underlying relationships between the measured variables, however, the sample size included in the analysis was not adequate and scored overall an “insufficient” rating with low-quality evidence.

### Internal Consistency

All three PROMs evaluated internal consistency, each scoring “high” for the quality of evidence. All questionnaires showed positive ratings, with Cronbach’s *α* scores ranging from 0.67 to 0.96, suggesting high interrelatedness among constituent outcome measure items. BREAST-Q studies 19^,^^20^ reported acceptable Cronbach’s *α* values (of ≥ 0.70) across the subscales (reconstruction module ranged from 0.88 to 0.96). There was an exception for surgical side effects within the EORTC QLQ-BRECON 23 questionnaire, which scored 0.67 for Cronbach’s *α*, below the acceptable threshold for internal consistency.

### Reliability

Reliability was assessed in all three PROMs. The quality of evidence for the measurement property varied, with only the BREAST-Q scoring as “high”-quality evidence. The intraclass correlation coefficient (ICC) was reported across all three PROMs. For BREAST-Q scale, reliability was supported by high Cronbach’s *α* values (> 0.80), high person separation indices (≥ 0.73), an ICC > 0.80, and appropriate item–total correlations (range of means 0.58–0.87). Test–retest reliability for all subscales of the BRECON-31 was good to excellent, with ICC showing excellent agreement (ICC = > 0.74) for six of the subscales and good to fair agreement for self-image, arm, intimacy, and nipple subscales. For EORTC QLQ-BRECON 23, test–retest reliability was good, with ICCs for multiitem scales ranging from 0.809 to 0.916 and single items from 0.728 to 0.905. However, the quality of evidence scores for reliability for BRECON-31 and EORTC QLQ-BRECON 23 were “very low” and “moderate,” respectively.

### Criterion Validity

Out of the three PROMs, only the BRECON-31 evaluated this measurement property.^15^ BRECON-31 used BREAST-Q as the reference standard (or gold standard) and performed well based on the level of concordance found between the two questionnaires. BRECON-31 showed excellent correlation (PCC = 0.76) for five of the subscales (satisfaction, self-conscious, arm concerns, appearance, and expectations).

### Hypothesis Testing for Construct Validity

Hypothesis testing for construct validity was assessed across all three PROMs, evaluating and demonstrating positive supporting evidence. BREAST-Q was compared with EORTC QLQ-BRECON 23, and hypotheses relating to correlations between BREAST-Q scales and other scales were widely supported through moderate correlations. BRECON-31 was compared with EQ-5D results. The EQ-5D showed moderate agreement with a summary score of the BRECON-31 (PCC = 0.50, *p* < 0.01), and utility ratings correlated moderately with BRECON-31 (PCC = 0.42, *p* < 0.001). Construct validity for the EORTC QLQ-BRECON 23 questionnaire was assessed using exploratory factor analysis (EFA). The EFA supported the phase 3 provisional six scale structure; all item–factor weights exceeded 0.4.

### Responsiveness

Of the three PROMs, only the EORTC QLQ-BRECON 23 evaluated this property. EORTC QLQ-BRECON 23 scored a sufficient overall rating and scored high for quality of evidence. Mean scale scores from baseline to 6 months were statistically significant (*p* < 0.001). For scales such as sexuality and surgical side effects, the effect sizes were small, 0.37 and 0.31, respectively.

### Information on Feasibility of PROMs

Table 7 summarizes the different aspects of feasibility evaluated for each PROM. BREAST-Q and EORTC QLQ-BRECON 23 were reported to be acceptable, comprehensible, and easy to complete by patients. The three PROMs differed slightly in the amount of time these took for patients to complete, due to differing numbers of items per subscale. The EORTC QLQ-BRECON 23 had the longest completion time for patients; however, this is designed to be used alongside two other questionnaires: EORTC QLQ-30 (cancer) and QLQ-BR23 (breast cancer). Both BREAST-Q and BRECON-31 have been validated in BR patients; however, EORTC QLQ-BRECON 23 has only been validated in patients undergoing BR for cancer and has not been validated in a risk-reducing population.

**Table 7:** Feasibility aspects of PROMs: BREAST Q, BRECON 31, and EORTC QLQ-BRECON23

Feasibility aspects	BREAST Q	BRECON 31	EORTC QLQ-BRECON 23
Patient comprehensibility	Patients found questionnaire to be acceptable, comprehensive, and clear	Not stated	Found to be acceptable for the majority of women and was quick and easy to complete
Domains/subscales and number of items (core outcomes are highlighted in bold)	Six subscales **Satisfaction with breasts (15 items)** Satisfaction with overall outcome (7 items) Satisfaction with information/care (15 items) **Psychosocial wellbeing (10 items)** Sexual wellbeing (6 items) **Physical wellbeing (chest and upper body) (11 items)** Total 69 items	Eight Subscales Self-image (4 items) Arm concerns (4 items) Intimacy (5 items) Satisfaction with outcome (4 items) Recovery (4 items) Self-consciousness (3 items) Expectations (4 items) Appearance (3 items) Total 31 items A nipple (4 items) and **abdominal subscale **are also used where applicable (10 items) giving maximum number of 45 items	Six subscales and three stand-alone items Surgery side effects (2 items) Sexuality (4 items) **Satisfaction breast cosmetic (6 items)** Satisfaction nipple cosmetic (2 items) Satisfaction with surgery (3 items) **Donor site symptoms (3 items)** Satisfaction with donor-site scar (single item) Loss of nipple (single item) Preserve/reconstruct nipple (single item) Total 23 items NB: This questionnaire is designed to be used alongside two other questionnaires: EORTC QLQ-30 (cancer) and QLQ-BR23 (breast cancer) Total 79 items
Completion time	Reconstruction module only: 10–15 min	5 min	20–30 min
Patient’s required mental and physical ability level	All content was targeted to sixth-grade reading level	The final items were refined for sixth-grade reading level according to Flesch-Kincaid, language and spelling according to Merriam-Webster online dictionary	
Ease of standardization	The BREAST Q scales are not considered valid for patient groups that were not represented in the development process		
Ease of score calculation	Acceptable	Easy scoring	Easy scoring
Copyright	Memorial Sloan Kettering Cancer Centre and Uni of British Columbia 2007	2012 Wiley Periodicals, Inc	EORTC
Cost of an instrument	No fee for use by academics	Not stated	No fee for use by academics
Required equipment	None	None	None
Regulatory agency’s requirement for approval	Local institutional ethics review board approval was obtained for 3 centers in the USA and Canada	Approved by the institutional review board of the University of Western Ontario	Ethical approval from the National Research Ethics Committee Northampton
No. of studies citing/using instrument questionnaire^a^	478	8	5
Other considerations	Validated in breast reconstruction patients	Validated in breast reconstruction patients	Only validated in patients undergoing breast reconstruction for cancer (not validated in risk-reducing population)

^a^As cited in Web of Science August 2019

## Discussion

This study is, to the best of the authors’ knowledge, the first to report a systematic review and critical appraisal of published studies reporting the measurement properties of PROMs developed for use in women undergoing BR using an updated COSMIN methodology.^11^ BR is performed to improve patients’ quality of life following mastectomy, and six key patient-reported outcome domains are included in the recently developed COS.^6^ It is vital that any PROM used to assess these important outcomes be robustly designed and validated if the results are to be meaningful. This review is the first necessary step to understand the performance of existing PROMs to inform instrument selection for patient-reported outcome domains in a BR CMS.

The systematic review identified 14 studies which included 6 different PROMs developed for use in a BR population. Of these, only three, BREAST-Q, BRECON-31, and EORTC QLQ-BRECON 23, were considered to have adequate content validity and were eligible for full measurement property assessment. All three instruments have been used to assess patient-reported outcomes in BR studies, but the most widely used and cited is BREAST-Q.^33^

BREAST-Q, BRECON-31, and EORTC QLQ-BRECON 26 all had thorough patient involvement in item generation and reduction, which has shown to be critical and to greatly increase the validity of BR PROMs.^22^

### Strengths and Limitations

This study has certain strengths and limitations. To the best of the authors’ knowledge, this is the first study that has used the recently updated COSMIN guidelines to assess the methodological quality of validation studies of BR PROMs. A validated and highly sensitive search strategy using published guidance from Terwee et al.^14^ was used to identify all potentially relevant studies, and three independent reviewers independently assessed the quality of each study (any disagreements resolved by a fourth reviewer), as recommended by COSMIN. The main limitation to this review is the assumption that, if validation studies of BR PROMs were not identified from the search, these had not been carried out. Therefore, the possibility of publication bias cannot be excluded. In addition, this review focused on PROMs developed in a BR population. However, there may be other instruments that may have value in this group (e.g., measures of self-esteem) but were not considered as these had not been developed or validated specifically in BR patients.

Critical appraisal was undertaken using the COSMIN checklist. This methodology has recently been developed and requires that PROM developers report in detail the methods used in the development and validation of their instrument. For PROMs developed before the introduction of COSMIN guidance, this information is often not reported in sufficient detail, if at all, and sometimes assumptions need to be made based on the information the author(s) have provided. Researchers developing PROMs in the future will need to follow COSMIN recommendations when reporting their studies to ensure complete reporting of study details and accurate interpretation of results.

### Further Work

The aim of this review was to identify robustly validated PROMs that could be recommended to measure the six key patient-reported outcome domains in the BR COS. The three PROMs identified in this review measure most of the key constructs with specific subscales that adequately address each domain. The domains of “normality” and “self-esteem,” however, are not constructs specifically included in any of the identified instruments, but both BREAST-Q and BRECON-31 include single items which reflect these domains. Further work is now required to determine whether patients feel that these items are adequate or whether work is needed to develop new PROMs in these areas.

Next steps will involve consensus work with key stakeholders to determine which of the three candidate PROMs should be recommended for use. This process involving a modified Delphi survey with over 100 professional stakeholders and face-to-face consensus meetings is already underway.^34^ Qualitative work with patients who have undergone BR surgery will also be needed to ensure that the selected PROMs are acceptable for this group.

## Conclusions

This systematic review identified three robustly developed and validated PROMs that could be recommended for use in future BR studies and inclusion in the CMS. Work is now required to determine which instrument should be routinely recommended for use to improve the quality and comparability of BR research and optimize its value for patients.

## Appendix: Search strategy for the systematic review of measurement properties of patient reported outcome measures in reconstructive breast surgery

“Breast”

1. Breast/
2. Breast.mp
3. OR/14-15
4. Limit 16 to English, humans

AND

“Reconstruction”

1. Reconstructive Surgical Procedures/
2. Surgical Flaps/
3. Surgery, Plastic/
4. Breast Implants/
5. Prostheses and implants/
6. Tissue Expansion Devices/
7. Reconstruct$.mp
8. Expan$.mp
9. Implant$.mp
10. Prosthe$.mp
11. Flap$.mp
12. Latissimus.mp
13. LD.mp
14. TRAM.mp
15. DIEP.mp
16. Plastic surg$.mp
17. Myocutaneous.mp
18. Myofascial.mp
19. Musculocutaneous.mp
20. Thoracodorsal.mp
21. TUG.mp
22. $GAP.mp
23. acellular dermal matri$.mp
24. mesh.mp
25. Strattice.mp
26. Surgimend.mp
27. AlloDerm.mp
28. BRAXON.mp
29. TiLOOP.mp
30. OR/1-29
31. Limit 30 (English, humans),

**AND**

COSMIN Measurement properties^1^ filter

1. (instrumentation or methods).sh.
2. (Validation Studies or Comparative Study).pt.
3. exp Psychometrics/
4. psychometr*.ti,ab.
5. (clinimetr* or clinometr*).tw.
6. exp “Outcome Assessment (Health Care)”/
7. outcome assessment.ti,ab.
8. outcome measure*.tw.
9. exp Observer Variation/
10. observer variation.ti,ab.
11. exp Health Status Indicators/
12. exp “Reproducibility of Results”/
13. reproducib*.ti,ab.
14. exp Discriminant Analysis/
15. (reliab* or unreliab* or valid* or coefficient or homogeneity or homogeneous or “internal consistency”).ti,ab.
16. (cronbach* and (alpha or alphas)).ti,ab.
17. (item and (correlation* or selection* or reduction*)).ti,ab.
18. (agreement or precision or imprecision or “precise values” or test-retest).ti,ab.
19. (test and retest).ti,ab.
20. (reliab* and (test or retest)).ti,ab.
21. (stability or interrater or inter-rater or intrarater or intra-rater or intertester or inter-tester or intratester or intra-tester or interobserver or inter-observer or intraobserver or intraobserver or intertechnician or inter-technician or intratechnician or intra-technician or interexaminer or inter-examiner or intraexaminer or intra-examiner or interassay or interassay or intraassay or intra-assay or interindividual or inter-individual or intraindividual or intra-individual or interparticipant or inter-participant or intraparticipant or intra-participant or kappa or kappa’s or kappas or repeatab*).ti,ab
22. ((replicab* or repeated) and (measure or measures or findings or result or results or test or tests)).ti,ab.
23. (generaliza* or generalisa* or concordance).ti,ab.
24. (intraclass and correlation*).ti,ab.
25. (discriminative or “known group” or factor analysis or factor analyses or dimension* or subscale*).ti,ab.
26. (multitrait and scaling and (analysis or analyses)).ti,ab.
27. (item discriminant or interscale correlation* or error or errors or “individual variability”).ti,ab.
28. (variability and (analysis or values)).ti,ab.
29. (uncertainty and (measurement or measuring)).ti,ab.
30. (“standard error of measurement” or sensitiv* or responsive*).ti,ab.
31. ((minimal or minimally or clinical or clinically) and (important or significant or detectable) and (change or difference)).ti,ab.
32. (small* and (real or detectable) and (change or difference)).ti,ab.
33. (meaningful change or “ceiling effect” or “floor effect” or “Item response model” or IRT or Rasch or “Differential item functioning” or DIF or “computer adaptive testing” or “item bank” or “cross-cultural equivalence”).ti,ab.
34. OR/ 1-33
35. Limit 34 to English

AND

Patient-reported outcome filter (University of Oxford)

1. (HR-PRO or HRPRO or HRQL or HRQoL or QL or QoL).ti,ab.
2. Quality of life.mp.
3. (health index* or health indices or health profile*).ti,ab.
4. Health status.mp.
5. ((patient or self or child or parent or carer or proxy) adj (appraisal* or appraised or report or reported or reporting or rated or rating* or based or assessed or assessment*)).ti,ab.
6. ((disability or function or functional or functions or subjective or utility or utilities or wellbeing or well being) adj2 (index or indices or instrument or instruments or measure or measures or questionnaire* or profile or profiles or scale or scales or score or scores or status or survey or surveys)).ti,ab.
7. OR/ 1-6
8. Limit 7 to English

Combine searches with AND.
